# Editorial: The exploration of low-dimensional nanoparticles for disease diagnosis and therapy

**DOI:** 10.3389/fbioe.2023.1227295

**Published:** 2023-07-03

**Authors:** Hua Yue, Zhen Liu, Shaohua Wang

**Affiliations:** ^1^ Institute of Process Engineering, Chinese Academy of Sciences (CAS), Beijing, China; ^2^ College of Life Science and Technology, Beijing University of Chemical Technology, Beijing, China; ^3^ Department of Biomedical Sciences, Ohio University Heritage College of Osteopathic Medicine, Athens, OH, United States; ^4^ Infectious and Tropical Disease Institute, Ohio University, Athens, OH, United States

**Keywords:** low-dimensional nanoparticles, antioxidant platform, antibacterial implants/therapy, anticancer therapy, tumor DNA biosensors, cell imaging, 2D nanomaterials

In addition to 3D particles, low-dimensional nanoparticles (LDNs), such as 0D dots, 1D tubes, and 2D sheets, have garnered tremendous interest in the fields of nanotechnology and pharmacology. To bridge knowledge gaps for LDNs and inspire further developments in their bio-applications, we organized this Research Topic. So far, the Research Topic presents nine selected, peer-reviewed contributions, including six original researches, and three mini-reviews.

The contributions introduce fabrication strategies for LDNs-based vesicles from 0D nanocluster/quantum dot, 1D rod-shaped particles, and 2D MXenes, sourced from organic polymer or inorganic carbon/metal materials. Detailed research or reviews mainly focus on diagnosis and disease therapy, such as antioxidant platforms, antibacterial implants/therapy, anticancer therapy, tumor DNA biosensors, and cell imaging.

There are two contributions regarding LDNs-based biosensor/imaging. One contribution from Yu et al. focuses on the *in-situ* reduction of gold nanoparticles-decorated MXenes-based electrochemical sensing platform for gene detection. The biosensor has a linear detection range of 10 fM–10 nM and a detection limit of 0.38 fM. It also efficiently distinguishes single base mismatched DNA sequences. The biosensor has been successfully used for the sensitive detection of KRAS gene G12D, which has excellent potential for clinical analysis. The other contribution from Du et al. refers to Carbon dots (CDs), which are prepared from corn stalk powder and used as fluorescent sensors for the selective detection of Fe3^+^ ions and biological cell imaging. The CDs have a low detection limit of 63 nM and high recognition for Fe3^+^ ions, as well as low cytotoxicity and desirable biocompatibility. The study supports the potential of converting agricultural waste into carbon nanomaterials.

There are three contributions regarding LDNs-based antimicrobial therapy. The first article discusses the use of ultra-small molybdenum-based nanodots for the treatment of periodontal disease, a local inflammatory disease that results in the destruction of tissue due to inflammation. The study introduces ultra-small molybdenum-based nanodots (MoNDs) with strong reactive oxygen species (ROS) scavenging capabilities, which is an effective strategy for oxidative stress-induced periodontal disease. Chen et al. demonstrate that MoNDs can alleviate periodontal inflammation by scavenging multiple ROS without any obvious side effects, thereby providing a candidate for the treatment of periodontal disease. The contribution from Hu et al. discusses the use of imidazole chloride ionic liquids for antibacterial activity. The authors comparatively evaluate the antimicrobial potency of imidazole chloride ILs (CnMIMCl) on *Staphylococcus aureus* (*S. aureus*). They discover that this nanozero material with long chain disrupted the bacteria membrane, causing the cytoplasm to flow out, and resulting in the fragmentation of the whole bacteria. The healing of skin abscesses was also accelerated within 12 days, demonstrating its potential as a candidate for the development of novel antibacterial agents. Another study in the Research Topic focuses on using gold nanoparticle decorated TiO_2_ nanotubes for sonodynamic therapy against peri-implant infections. Sun et al. develop an antibacterial implant surface based on Au nanoparticle-modified TiO_2_ nanotubes (AuNPs-TNTs). The study shows that the as-proposed AuNPs-TNTs exhibit significantly enhanced antibacterial activity under a simple ultrasound treatment, offering a way to design the surface of an artificial implant coating for resolving the bacterial infection-induced failure of dental implants.

The last original research is a study on the preparation strategy of one-dimensional poly (lactic-co-glycolic acid) particles for paclitaxel delivery. By using the emulsion solvent evaporation method with Na_2_HPO^4^ and sonication, the yield of nanorods in the optimal formula was 99%, and the aspect ratio was 5.35 ± 2.05. The size, shape, and aspect ratio of the nanoparticles could be controlled by manipulating process parameters, with surfactant PVA concentration being the most important factor. In addition, the anti-cancer drug paclitaxel could be successfully encapsulated in these nanorods. Although *in vitro* assessment has not been performed in detail, the present study provides an important avenue for fabricating non-spherical LDN using degradable materials, which endow the transform feasibility for further application in potential cancer therapy.

The Research Topic also includes three mini-reviews on LDNs as an emerging platform for cancer diagnosis and disease therapy. One review from Cui et al. summarizes the distinctive physicochemical capabilities of LDNs favored by biomedical applications. It emphasizes a multimodal nano-platform and relative applications in the imaging, diagnosis, and treatment of cancerous diseases. The review from Tang et al. highlights the advantages of 0-, 1-, and 2-dimensional carbon materials that help protect cells against oxidative stress. It also discusses their challenges and perspectives in biomedical fields and further clinical usages. Another review from Chen et al. focuses on the use of 2D nanomaterials (e.g., DNA origami, germanene, and MXene) for acute kidney injury (AKI) treatment. The review also highlights the challenges and future opportunities in the field, aiming to provide insights and theoretical support for the development of novel 2D nanomaterials for AKI treatment.

Overall, the Research Topic provides a comprehensive overview of recent research advances in nanomedicine and nanotechnology. The original work and reviews included in the Research Topic demonstrate the enormous potential of LDNs for a wide range of biomedical applications. The articles also provide insight into the current challenges and future opportunities associated with the development and application of these advanced materials. This Research Topic is expected to be of great interest to researchers and professionals working in the fields of bio-sensing, nanotechnology, and pharmacology.

